# Split-Hopkinson Pressure Bar Test of Silicone Rubber: Considering Effects of Strain Rate and Temperature

**DOI:** 10.3390/polym14183892

**Published:** 2022-09-17

**Authors:** Xintao Zhu, Xiaojing Zhang, Wenjin Yao, Wenbin Li

**Affiliations:** ZNDY of Ministerial Key Laboratory, Nanjing University of Science and Technology, Nanjing 210094, China

**Keywords:** silicone rubber, split-Hopkinson pressure bar, strain rate, temperature, differential scanning calorimetry

## Abstract

Using a split-Hopkinson pressure bar test instrument with a temperature control device, in this work, silicone rubber was tested at different temperatures (−40 °C–200 °C) and different strain rates (1.2 × 10^3^ s^−1^–7.2 × 10^3^ s^−1^). The results showed that the dynamic mechanical properties of silicone rubber were strain-rate sensitive at different temperatures and the yield strength of the silicone rubber increased with an increase in the strain rate. At a higher strain rate, silicone rubber showed temperature sensitivity. With a decrease in the strain rate, the influence of temperature on silicone rubber gradually decreased. Differential scanning calorimetry analysis showed that silicone rubber had good thermal stability at high temperatures. When the temperature was as low as −40 °C, the silicone rubber underwent a glass transition, showing the characteristics of brittle materials.

## 1. Introduction

Coating high explosives with a protective layer can improve their shock absorption and heat insulation ability as well as their stability [1]. As a type of buffer layer, a coating layer with silicone rubber as the main component is widely used in the combustion chambers of rocket engines and also in ground−penetrating projectiles. Silicone rubber is a synthetic material with excellent properties, where the main chain of silicone rubber is composed of alternating silicon and oxygen atoms, and the silicon atoms are connected by two organic side groups. Therefore, silicone rubber has the characteristics of both organic and inorganic materials, offering excellent thermal stability and aging resistance. High− and low−temperature resistances are the best among general rubber, offering excellent weather resistance, good insulation, and strong resistance to nitroglycerin migration. Silicone rubber has good compatibility with aluminum, steel, explosives, and other materials, and is a type of low−smoke coating material with great development potential [2]. As a coating material, silicone rubber needs to be able to bear certain stress and deformation during its transportation, storage, and use. The performance of silicone rubber under impact under high− and low−temperature conditions will inevitably affect the stability and safety of internal explosives. The mechanical properties of rubber materials can be closely related to the ambient temperature, strain history, and loading rate. Therefore, studying the dynamic mechanical properties of silicone rubber materials under different strain rates and temperatures is of great significance.

Split-Hopkinson pressure bar (SHPB) technology has been widely used to characterize the dynamic characteristics of materials by providing the stress–strain curves of materials, especially the flow stress under large deformation with strain rates of 10^2^ s^−1^ to 10^4^ s^−1^ [3,4,5,6,7,8,9]. Miao et al. [10] used the SHPB test to measure the dynamic elastic modulus of polymer materials under a high−strain loading rate. By comparing with traditional SHPB experimental results, the researchers found that the vertical SHPB could provide more accurate measurements. Li et al. [11,12] measured the dynamic mechanical properties of high−damping rubber through SHPB experiments and studied the variation law of dynamic compression performance with the strain rate; they found that the strength and energy absorption capacity of high−damping rubber materials increased with an increase in the strain rate. Jiang et al. [13] proposed an optimization design method for a polymer SHPB system pulse shaper for soft material testing based on wave propagation analysis and determined the optimal incident waveform of a viscoelastic specimen. Pang et al. [14] carried out uniaxial compression experiments on three rubber materials with different wave impedance values in a large range of strain rates. The results showed that the hardening effect gradually increased with an increase in the strain rate under dynamic loading. Zhou et al. [15] studied the stress–strain curve, energy absorption performance, and wave transmission performance of metal rubber under different strain rates on an SHPB device. The results showed that the metal rubber had an excellent energy absorption capacity and impact resistance. Lu et al. [16] tested silicone rubber using SHPB under three different interfacial friction conditions (lubrication, drying, and bonding) and the results showed that interfacial friction significantly increased the flow stress. In addition, many researchers have studied the effect of temperature on the mechanical behavior of rubber [17,18,19,20]. Trivedi et al. [21] studied the relationship between the response of neoprene under different strain rates and temperatures and proposed a simple model to describe the behavior of materials using the principle of time–temperature superposition (TTS). Zhou et al. [22] conducted Hopkinson rod impact tests on rubber materials at different temperatures and strain rates and combined with viscoelastic and hyper−elastic theories, established a large strain nonlinear thermal viscoelastic hyper−elastic constitutive model that could reflect the temperature and strain−rate effects of rubber materials.

Although some researchers have studied the dynamic mechanical properties of silicone rubber at different temperatures, the experimental temperature range was narrow, and high and low temperatures were usually not combined for comparison. To fully understand the effects of different strain rates and temperatures on the mechanical behavior of silicone rubber, in this study, dynamic compression experiments were carried out at high and low temperatures, and the stress–strain response curves of silicone rubber at different temperatures and strain rates were obtained.

## 2. Materials and Methods

### 2.1. SHPB Experimental Device and Principle

The SHPB test is considered the main experimental means for obtaining the stress–strain relationship of materials in a strain−rate range of 100 s^−1^–10,000 s^−1^. This avoids the difficulty of directly measuring the dynamic large deformation of the specimen. By measuring the small deformation of the waveguide rod, the stress–strain relationship of specimens with high−speed large deformations can be deduced according to the stress−wave theory. SHPB is an effective experimental method for studying the mechanical properties of materials at high strain rates and has been widely used worldwide. The SHPB test method is based on two basic assumptions (one−dimensional stress wave assumption and stress uniformity assumption): (1) During the loading process, the axes of the impact rod, incident rod, as well as the specimen and transmission rod in the SHPB device, are completely coincident and the stress pulse propagating in the compression rod consists of a completely one−dimensional stress wave; thus, the experimental conditions fully meet the one−dimensional stress wave assumption. (2) At any time throughout the loading process, the internal stress and strain of the specimen are uniform, and the experimental conditions fully meet the assumption of uniform stress.

**Figure 1:**
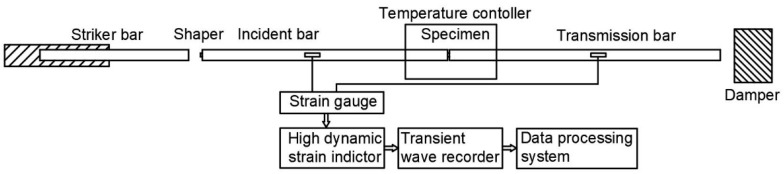
Schematic of the SHPB system with temperature controller.

In this work, experimentation was carried out on an SHPB experimental device with a temperature control box at the Nanjing University of Technology and the equipment diagram is shown in Figure 1. The length of the impact rod was 0.2 m and the incident and transmission lengths were 1.5 m and 2 m, respectively. The impact rod, incident rod, and transmission rod had a diameter of 14.5 mm with a density of 2.81 g/cm^3^. To reduce friction in the test, both ends of the test piece were coated with grease. During the experiment, at least three experiments with good repeatability were carried out under each strain rate and the three effective curves were averaged to obtain the stress–strain curve under the strain rate.

Because the transmission signal of silicone rubber was weak, the semiconductor strain gauge was used to replace the traditional resistance strain gauge to measure the transmission signal in the experiment. During the experiment, the material was placed in an incubator, cooled by liquid nitrogen during low−temperature testing, and heated by a resistance wire during high−temperature testing. Due to the low thermal conductivity of rubber, the test piece was kept warm for at least 50 min before the experiment. In the test, the strain gauge of the transmission rod was glued 1 m away from the rod specimen interface to reduce the influence of the heat source on the signal of the semiconductor strain gauge. In a temperature range of –40 °C to 200 °C, the modulus of the aluminum rod did not change and the influence of the temperature gradient field on the wave propagation could be ignored.

**Figure 2:**
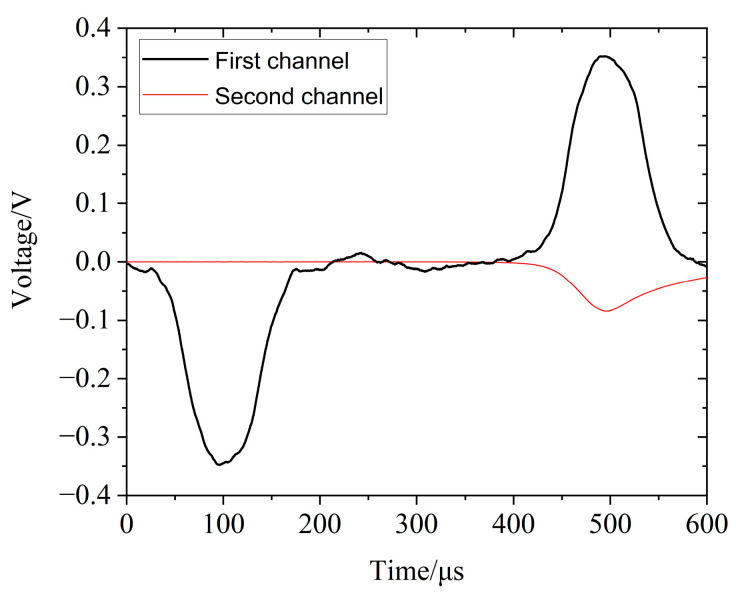
Typical original signal waveform of silicone rubber.

Figure 2 shows the original waveform of the silicone rubber obtained in the SHPB experiment, where channel 1 recorded the incident wave and the reflected wave and channel 2 recorded the transmitted wave. The wave velocity in the silicone rubber material was low, and even for a very thin sample, the time required for the wave to propagate in the sample was relatively long. Generally, the front peak of the stress wave would propagate back and forth in a sample more than three times, allowing the stress in the sample to reach a basically uniform state, thus meeting the assumption of uniform stress in the specimen in the SHPB one−dimensional stress wave theory [23]. Therefore, for a long time, the specimen would be in a state of uneven stress and the assumption of stress uniformity often used in data processing would be no longer tenable, according to the premise of the split-Hopkinson pressure bar experiment method. As a result, the SHPB test for silicone rubber materials would require waveform shaping [24,25]. 

**Figure 3:**
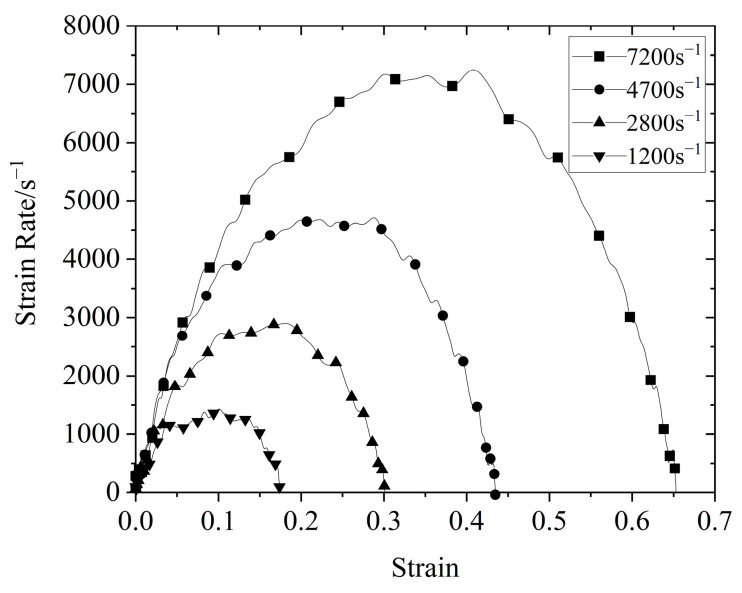
Strain−rate strain curve of silicone rubber at room temperature.

Proper waveform shaping can not only homogenize the internal stress of the loaded sample but can also realize the constant strain−rate deformation of the sample. For soft material SHPB experiments, brass and rubber are most commonly used among the many optional shaper materials. The size of the shaper differs due to the characteristics of the tested material; thus, it would have to be designed. According to the research results of Lu et al. [26,27], rubber with a diameter of 4 and a thickness of 1.5 mm was selected as the shaper in this study. Figure 3 shows the strain−rate strain curve of silicone rubber obtained through the SHPB experiment. As shown in Figure 3, it was clear that constant strain-rate loading was achieved in the experiment.

### 2.2. Experimental Sample

**Figure 4:**
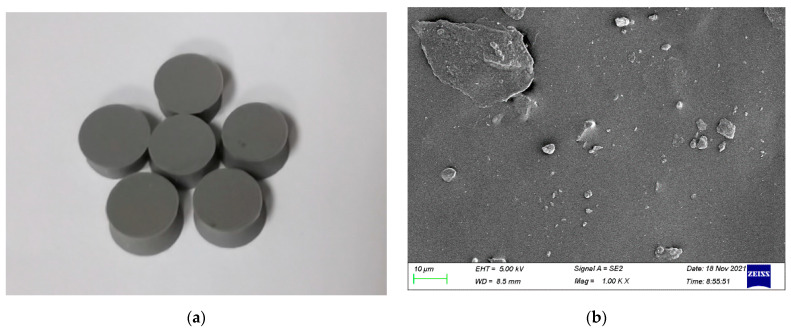
Silicone rubber sample: (**a**) The apparent morphology of the silicone rubber; (**b**) SEM micrograph of the silicone rubber.

The specimen in this experiment was room−temperature vulcanized silicone rubber provided by the 53rd Research Institute of China Electronics Technology Group Corporation, as shown in Figure 4. The room−temperature vulcanized silicone rubber was prepared using silicone rubber as the main raw material, to which an optimized crosslinking agent, hardener, and vulcanizing agent were added. The material has good heat resistance, compatibility, few components, good fluidity, can be vulcanized at room temperature, and is suitable for casting. The chemical structure of room−temperature vulcanized silicone rubber is light−terminated polydimethylsiloxane, which is easy to rotate freely. The molecule is very soft and easy to curl into a helical open structure with six to eight silicon–oxygen bonds as repeating structural units. This helical structure makes the two methyl groups shield the silicon–oxygen bond, and the polarity of the silicon and oxygen atoms cancel each other out, making the whole molecule non−polar. The attraction between the molecular chains is very small and it is easy to slide so its cohesive energy density is very low and its mechanical strength is relatively low. Selecting a specimen with a smaller thickness can shorten the time of the stress wave propagation once, allowing the specimen to quickly reach stress balance and ensuring the uniform deformation of the specimen during the experiment. A short cylindrical specimen with a diameter of 5 mm, a thickness of 2.5 mm, and a density of 1.084 g/cm^3^ was selected for this experiment.

## 3. Results and Discussion

### 3.1. Failure Characteristics

**Figure 5:**
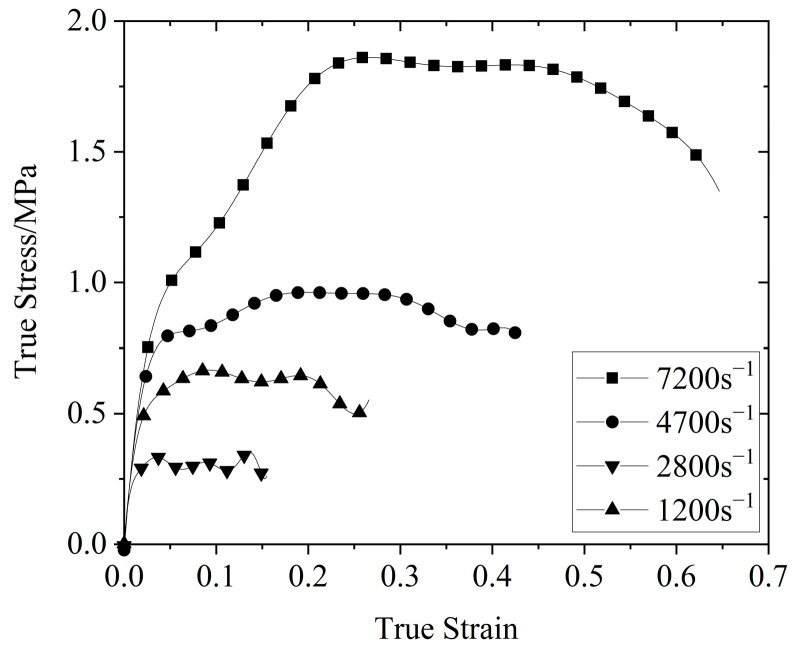
Stress–strain curve of silicone rubber at room temperature (26 °C) and different strain rates.

Figure 5 shows the stress–strain curves of the silicone rubber at four different strain rates at room temperature (26 °C). As shown in Figure 5, the yield strength and yield strain of the silicone rubber increased with an increase in the strain rate, showing obvious rate−related characteristics, and the initial section of the stress–strain curve basically coincided. When the strain exceeded 0.015, the strain−rate effect significantly increased. The stress–strain curve of silicone rubber could be roughly divided into three stages: the initial linear rise stage, the yield stage, and the final stress unloading stage. In the small strain range during the initial stage of loading, the stress increased sharply with the strain and the trend was generally linear. This stage consisted of the initial rising stage, which was caused by the strengthening effect as a result of the rapid increase in the internal strain rate of the specimen during the initial stage of loading. When the stress reached the yield strength under the corresponding strain rate, the material yielded, and the stress–strain curve entered the yield stage. With an increase in the strain, the stress did not increase. After loading, the test piece started to unload, which consisted of the unloading section of the curve.

**Figure 6:**
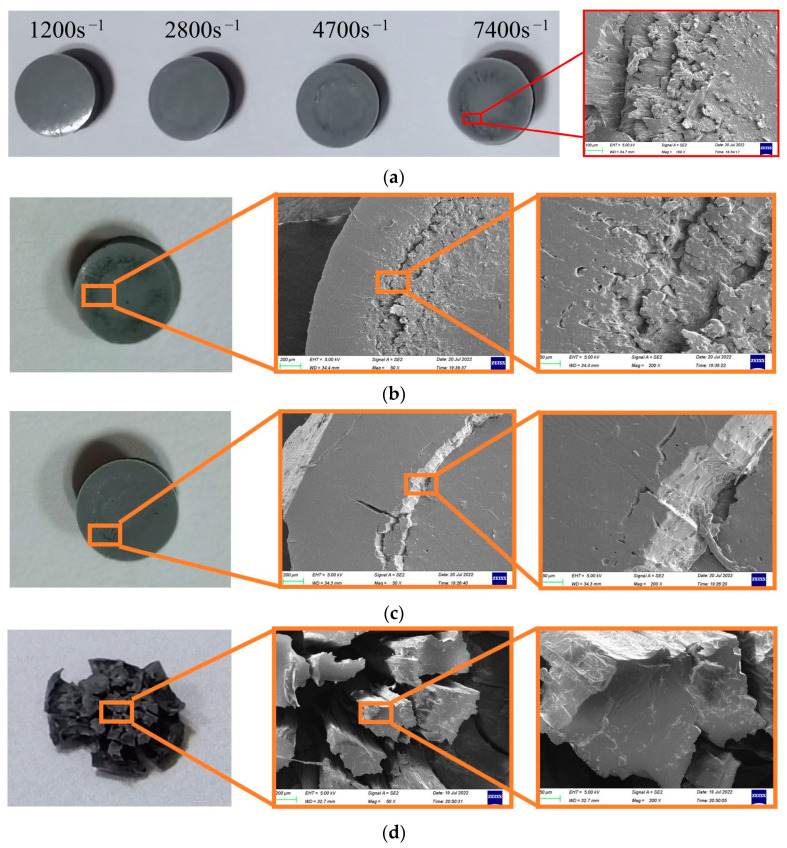
Damage morphology of silicone rubber after impact: (**a**) Damage morphology of the silicone rubber at the same temperature (26 °C) and different strain rates; (**b**) Typical damage micromorphology of silicone rubber at a low temperature (200 °C, 7400 s^−1^); (**c**) Typical damage micromorphology of silicone rubber at a low temperature (−40 °C, 7400 s^−1^); (**d**) Damage morphology of the silicone rubber after impact at higher speeds (26 °C).

Figure 6 shows the morphology of the silicone rubber sample after experimentation, and Figure 6a shows the morphology of the silicone rubber specimen after impact at different rates at room temperature (26 °C), as well as the SEM photos of the damaged area. We observed that the damaged line was the macroscopic manifestation of the local damage and cracking of the material. As shown in Figure 6a, when the strain rate was not greater than 2800 s^−1^, there was no damage to the surface of silicone rubber after impact. When the strain rate reached 4700 s^−1^, a circle of obvious damage appeared on the surface of the material after impact. The damage degree increased with the increase in the strain rate.

Figure 6b is an SEM diagram of the damaged line of silicone rubber after impact at high temperatures. Figure 6b shows the damage morphology of silicone rubber after impact at high temperatures, which was consistent with that at normal temperatures, and the end face was damaged along the damaged line. The damage was uneven and multi−layer annular tear lines can be seen. The cracks at the damage all developed along the diameter direction of the end face. The SEM diagram of the silicone rubber after impact at low temperatures in Figure 6c shows that the damaged line at the end face was neatly split, unlike the multi−layer damage line at normal and high temperatures. At low temperatures, there were almost no micro−cracks on the damaged line after impact, showing the characteristics of brittle materials.

The residual thickness of the sample in the damaged line area was small, showing residual deformation after compression loading, and the residual thickness of the part outside the line was relatively large. This phenomenon showed good repeatability in the experiments under the same loading conditions. Under different loading conditions, the radius and damage degree of the damaged line area were different. When the impact load strength was small, no damage line was observed. With an increase in the load intensity, the location of the damaged line shrank from the outside to the inside. Visually, the color of the line changed from light to deep and the damage degree of the damaged area changed from weak to strong. Finally, disintegration and destruction of the material occurred within a certain range in the center, and the local material morphology changed from an overall structure to a flocculent structure, as shown in Figure 6d. Similar results were also reported by Lin et al. [28].

### 3.2. Stress–Strain Curve

Amorphous polymers, such as silicone rubber, exhibit three mechanical states with changes in temperature, namely, the glassy state, highly elastic state, and viscous flow state. Their mechanical properties are also clearly dependent on the time and temperature, with time dependence also showing the dependence of the strain rate. The mechanical response of polymer materials is different under different strain rates, and the transition of amorphous polymers between glassy and highly elastic states is also known as the glass transition state, whereas the corresponding transition temperature can be referred to as the glass transition temperature for short. When the temperature was above the glass transition temperature, the silicone rubber exhibited viscoelasticity. When the temperature was below the glass transition temperature, if the action rate of the force was relatively fast, it was brittle and only had an instantaneous elastic response. If the action rate of the force was quite slow, it showed viscoelasticity.

**Figure 7:**
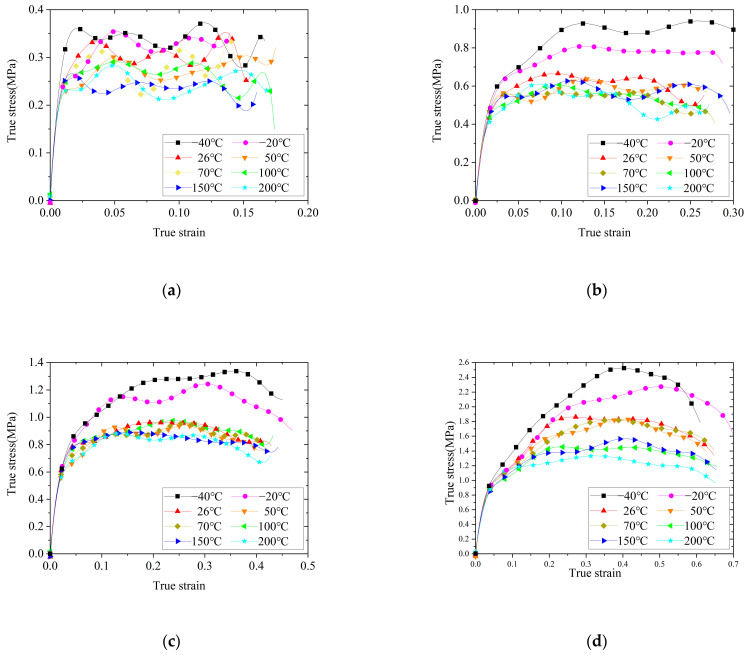
True stress–strain curves of silicone rubber under the same strain rate and different temperatures: (**a**) 1200 s^−1^ (**b**) 2800 s^−1^ (**c**) 4700 s^−1^ (**d**) 7200 s^−1^.

The stress–strain curves under the same strain rate and different temperatures are shown in Figure 7, which indicated that the stress–strain behavior of silicone rubber was clearly affected by the temperature. When the strain rate was 1200 s^−1^, the silicone rubber was less affected by the temperature. When the strain rates were 2800 s^−1^ and 4700 s^−1^, the silicone rubber was clearly affected by low temperatures, and the yield strength increased with decreased temperatures; however, it was less affected by high temperatures. When the strain rate was 7200 s^−1^, the silicone rubber was clearly affected by high and low temperatures. Within the experimental temperature range (−40 °C to 200 °C), the yield strength of silicone rubber decreased with an increase in the temperature. When the temperature was −40 °C, with an increase in the strain rate, the plateau section of the stress–strain curve of silicone rubber gradually decreased after yielding. When the strain rate increased to 7200 s^−1^, the stress–strain curve showed the characteristics of brittle materials, and we speculated that −40 °C was close to the glass transition temperature of silicone rubber. The glass transition behavior of Cr rubber at low temperatures was also reported by Wang et al. [17].

**Figure 8:**
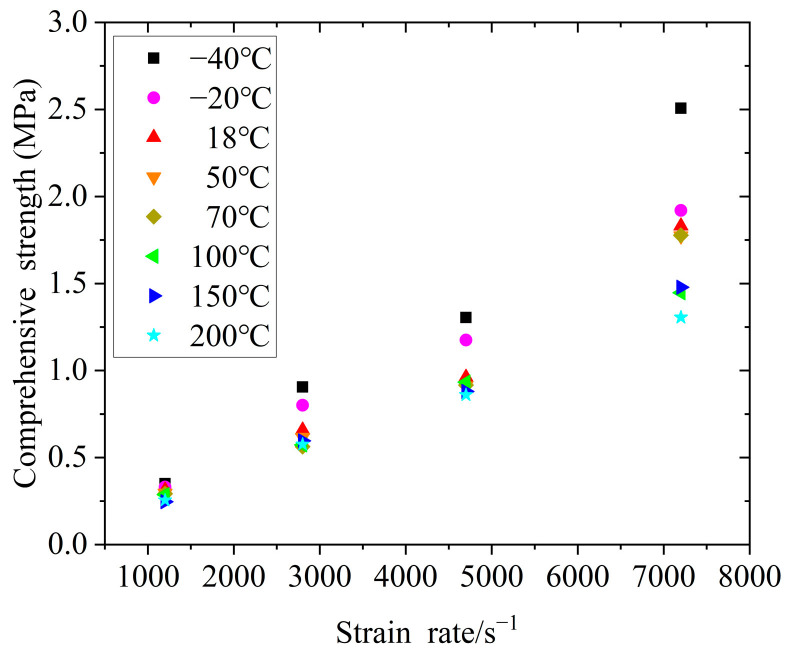
Relationship between the yield strength and strain rate.

**Figure 9:**
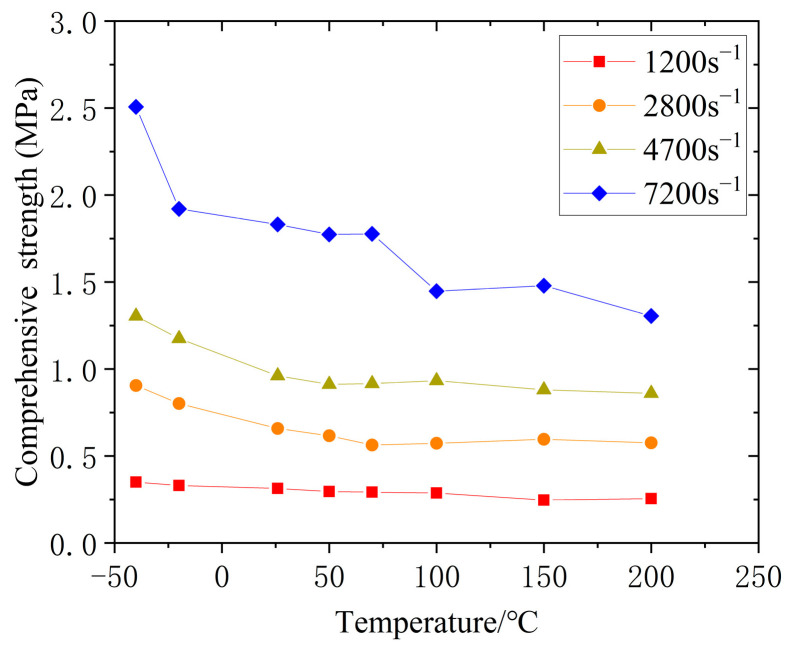
Relationship between yield strength and temperature.

Figure 8 and Figure 9 show the curves of the yield strength of silicone rubber with the strain rate and temperature, respectively, where the strength change of the silicone rubber under impact loading had a certain regularity. Specifically, within the range of experimental strain rates and temperatures, the yield strength of silicone rubber increased with an increase in the strain rate, reflecting the significant correlation between the stress–strain behavior and strain rate. As shown in Figure 9, when the strain rate was lower than 7200 s^−1^, high temperatures had little effect on the dynamic yield strength. When the strain rate reached 7200 s^−1^, high temperatures affected the yield strength, where the yield strength decreased with an increase in the temperature. Under different strain rates, low temperatures had a great influence on the dynamic yield strength, where the yield strength increased sharply with a decrease in the temperature. Similar behaviors of the dynamic compression response of rubber materials at low temperatures were reported by Lee et al. [20] and Trivedi et al. [21]. Thus, the higher the strain rate, the greater the effect of low temperatures on the yield strength of silicone rubber.

## 4. DSC Analysis of Silicone Rubber

Differential scanning calorimetry (DSC) can measure the relationship between the power difference and temperature difference between a sample and reference at a programmed temperature. The curve recorded by a differential scanning calorimeter is called the DSC curve, which takes the rate of the heat absorption or heat release of the sample, namely, the heat flow rate DH/DT as the ordinate and the temperature T or time t as the abscissa. DSC can measure various thermodynamic and kinetic parameters such as the specific heat capacity, reaction heat, transition heat, phase diagram, reaction rate, crystallization rate, polymer crystallinity, and sample purity. The DSC method offers a wide temperature range (–175–725 °C), high resolution, and less sample consumption, and can obtain all types of information needed in a short time, making it suitable for the analysis of inorganic substances, organic compounds, and drugs.

According to the verification method specified by the verification regulation of a jjg936−2012 differential scanning calorimeter, a DSC−25 (TA−instrument) was used to analyze the silicone rubber. Indium was placed in a standard aluminum crucible as the reference material for thermal analysis, where the flow rate of nitrogen was 50 mL/min, the heating rate was 10 °C/min, and the temperature range was –90 °C–200 °C. In addition, the ambient temperature of the experiment was 26 °C, the surroundings were well ventilated, and there were no strong vibrations or electromagnetic interference.

**Figure 10:**
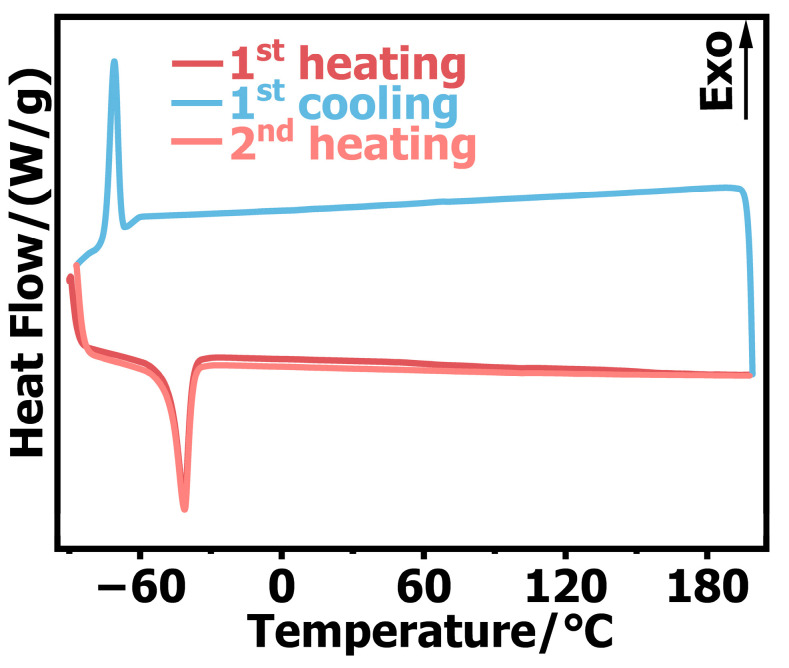
DSC curves of silicone rubber.

The DSC experimental curve is shown in Figure 10, where two heating processes and one cooling process were carried out in the experiment. The experimental data and calculation results are shown in Table 1, where T_0_ denotes the initial exothermic/endothermic temperature and T_p_ is the exothermic and endothermic peak temperatures. We observed an endothermic peak in the first round of heating and a corresponding exothermic peak in the second round of cooling, which indicated that the crystalline phase of silicone rubber changed at –40 °C. There was also an endothermic peak in the second round of heating, and the T_0_, T_p_, and enthalpy changes of the endothermic peak were almost the same as those in the first round of heating, which indicated that no decomposition reaction occurred in the silicone rubber throughout the heating and cooling processes. In the high−temperature process, the silicone rubber did undergo endothermic and exothermic reactions, indicating that the silicone rubber showed stable performance at high temperatures and the internal structure did not change. Comparing Figure 7, Figure 8 and Figure 9, the DSC analysis results confirmed that silicone rubber underwent a glass transition at temperatures as low as –40 °C, thus showing brittle material characteristics under the high−speed impact (7200 s^−1^). This is consistent with the glass transition temperature of silicone rubber composites reported by Rana et al. [29] and Wang et al. [30].

## 5. Conclusions

In this work, an SHPB experimental system was used to test silicone rubber at different temperatures (–40 °C–200 °C) and high strain rates (1 × 10^3^ s^−1^–7 × 10^3^ s^−1^), and accurate dynamic compression test data were obtained. Combined with the DSC analysis results, the effect of temperature on silicone rubber was further studied and the main conclusions were as follows.

(1) Silicone rubber was sensitive to the strain rate. In the range of experimental temperatures and strain rates, the yield strength of silicone rubber increased with an increase in the strain rate.
(2) Silicone rubber was sensitive to low temperatures but was only sensitive to high temperatures at high strain rates. When the temperature was lower than room temperature, the yield strength increased with a decrease in the temperature. When the temperature was higher than room temperature, the yield strength decreased with an increase in the temperature at a high strain rate (7200 s^−1^). When the strain rate was less than 7200 s^−1^, high temperatures did not affect the yield strength and the yield strain of the silicone rubber was hardly affected by the temperature.
(3) The DSC results showed that the internal crystalline phase of silicone rubber changed at –40 °C and glass transition occurred. Under the condition of a high strain rate, the silicone rubber showed brittle material characteristics.

In future work, we plan to conduct dynamic mechanical property experiments on aged silicone rubber to provide more technical support for the service conditions of silicone rubber as a coating.

## Figures and Tables

**Table 1:** DSC experimental data and calculation results of silicone rubber.

Experimental Process	T_0_ (°C)	T_P_ (°C)	Enthalpy (J/g)
First heating	−58.274	−40.815	17.794
First cooling	−70.888	−67.126	/
Second heating	−58.292	−40.821	17.782

## Data Availability

The data presented in this study are available on request from the corresponding author.
